# Normal GCAPs partly compensate for altered cGMP signaling in retinal dystrophies associated with mutations in *GUCA1A*

**DOI:** 10.1038/s41598-019-56606-5

**Published:** 2019-12-27

**Authors:** Daniele Dell’Orco, Giuditta Dal Cortivo

**Affiliations:** Department of Neurosciences, Biomedicine and Movement Sciences, Section of Biological Chemistry, University of Verona, I-37134 Verona, Italy

**Keywords:** Biochemistry, Neurochemistry, Visual system

## Abstract

Missense mutations in the *GUCA1A* gene encoding guanylate cyclase-activating protein 1 (GCAP1) are associated with autosomal dominant cone/cone-rod (CORD) dystrophies. The nature of the inheritance pattern implies that a pool of normal GCAP proteins is present in photoreceptors together with the mutated variant. To assess whether human GCAP1 and GCAP2 may similarly regulate the activity of the retinal membrane guanylate cyclase GC-1 (GC-E) in the presence of the recently discovered E111V-GCAP1 CORD-variant, we combined biochemical and *in silico* assays. Surprisingly, human GCAP2 does not activate GC1 over the physiological range of Ca^2+^ whereas wild-type GCAP1 significantly attenuates the dysregulation of GC1 induced by E111V-GCAP1. Simulation of the phototransduction cascade in a well-characterized murine system, where GCAP2 is able to activate the GC1, suggests that both GCAPs can act in a synergic manner to mitigate the effects of the CORD-mutation. We propose the existence of a species-dependent compensatory mechanism. In murine photoreceptors, slight increases of wild-type GCAPs levels may significantly attenuate the increase in intracellular Ca^2+^ and cGMP induced by E111V-GCAP1 in heterozygous conditions. In humans, however, the excess of wild-type GCAP1 may only partly attenuate the mutant-induced dysregulation of cGMP signaling due to the lack of GC1-regulation by GCAP2.

## Introduction

The absorption of photons by visual pigments in retinal rod and cone cells triggers a complex signaling cascade known as phototransduction, which results in the electrical response of the cell and generates the visual stimulus^1^. A variety of protein-protein, protein-nucleotide and protein-ion interactions finely regulates the cascade^2^, whose dynamics depend on the interplay between Ca^2+^ and cyclic guanosine monophosphate (cGMP), the second messengers involved in the signaling process^3,4^. The core of the Ca^2+^/cGMP signaling unit resides in the complex formed by the membrane retinal guanylate cyclase (GC1, also called ROS-GC1 or GC-E being the main cyclase in photoreceptor outer segments^5^) and guanylate cyclase-activating proteins (GCAPs). This supramolecular machinery ensures a precise control of the cGMP synthesis by GC1 as a consequence of the level of intracellular free Ca^2+^, which drops from few hundred nanomolar in the dark to below 100 nM in the light^1,6^. Several GCAP isoforms exist in various species. In mouse^7^ and in bovine^8,9^ photoreceptors both GCAP1 and GCAP2 have been shown to regulate the target GC1, while in humans so far only GCAP1 has been directly shown to regulate the activity of GC1^10,11^.

GCAPs are neuronal calcium sensors that detect subtle changes in Ca^2+^ concentration and adopt specific conformations required for controlling the activity of the target GC1, *per se* unable to respond to Ca^2+^. At high [Ca^2+^], GCAPs adopt a Ca^2+^-loaded state that inhibits GC1 activity, but following the drop in intracellular [Ca^2+^] during the light activation of the cascade, they switch to a Mg^2+^-bound conformation^12–15^ that stimulates GC1 to rapidly restore dark-adapted cell conditions by enhancing the synthesis of cGMP^3,16^.

To date, 20 missense mutations have been found in the *GUCA1A* gene encoding GCAP1, which have been associated with cone (COD) or cone-rod (CORD) dystrophies, severe forms of retinal dystrophy characterized by central vision loss, impaired color vision and photophobia^17–19^. No COD/CORD mutation in the gene encoding GCAP2 is known to date. When studied in reconstituted *in vitro* systems, most of the point mutations in *GUCA1A* resulted in GCAP1 variants that constitutively activate the GC1 over the physiological range of [Ca^2+^]^10,20–25^. The Y99C-GCAP1 mutation was the first one to have been discovered^24^, moreover it was the first one to be studied in a transgenic mouse line^26^. Other transgenic mouse lines were then generated to study similar COD/CORD-related phenotypes associated with mutations in GCAP1, namely the E155G-GCAP1^27^ and L151F-GCAP1^28^ models. The *in vivo* studies showed that the COD-related mutant caused photoreceptor degeneration due to an elevated [Ca^2+^] in the rod outer segment. However, rod photoresponses from the Y99C-GCAP1 mice showed relatively little changes especially at bright flashes, indicating a partly preserved Ca^2+^-regulated cGMP synthesis. Interestingly, the rate of photoreceptor cell loss increased with the level of Y99C-GCAP1 expression^26^.

For bovine and murine photoreceptors, it is well established that GCAP1 and GCAP2 are both capable of activating and inhibiting the target GC1 at similar levels in the physiological range of [Ca^2+^], however the two GCAPs differ from one another in some features: (i) GCAP1 has a slightly lower affinity for Ca^2+^ compared to GCAP2^12^; (ii) GCAP1 triggers the activation of GC1 earlier than GCAP2, that is at dimmer light conditions and/or when intracellular Ca^2+^ is still relatively high^3^; (iii) both GCAPs are myristoylated, but the myristoylation seems to exert a more prominent role for GCAP1 compared to GCAP2 in GC1 activation^9^. Moreover, in bovine cells the cellular concentration of GCAP1 and GCAP2 sums to the cellular concentration of GC1^9^. While it is clear that both GCAPs can regulate GC1 over the narrow physiological window of intracellular Ca^2+^, quite different mechanisms have been proposed for the regulation. Some studies support distinct binding interfaces between GC1 and GCAP1/GCAP2, the first GCAP being located at the juxtamembrane region and the second prevalently bound to the catalytic-C terminal domain^29–31^, thus allowing the binding of GCAP1 and GCAP2 to either the same or distinct GC1 molecules, but always in different interfaces On the other hand, other studies found a partly overlapped GC interface for GCAP1 and GCAP2^32,33^, suggesting a competition between GCAP1 and GCAP2 for GC1 activation in mouse cones^34^ as well as in rods. Overall, GCAPs seem to operate in a “calcium-relay” mode, thus acting simultaneously on either the same or distinct GC1 molecules to induce gradual responses in terms of cGMP synthesis triggered by small changes in intracellular [Ca^2+^]^3,35^. Their slightly different GC1-regulatory properties and Ca^2+^-sensitivity would therefore be needed to achieve the complex temporal dynamics necessary for finely tuning the enzymatic activity of GC1 over the broad range of light stimuli typical of photoreceptors.

COD and CORD linked to mutations in *GUCA1A* are associated with autosomal dominant (ad) inheritance pattern, thus implying that one half of the GCAP1 molecules in the overall GCAP pool would carry the mutation in affected photoreceptors. Under the assumption that no other alteration beside the missense mutation occurs, a normal amount of GCAP2 molecules would be therefore still present, together with one half of normal GCAP1. In this work we present the results of biochemical assays of human GC1 catalytic activity performed with different amounts of human wild-type (WT)-GCAP1, GCAP2 and E111V-GCAP1, a recently discovered variant associated with a severe form of CORD^10^. Interestingly, we found that human GCAP2 is uncapable of activating GC1 and therefore does neither co-act nor compete with GCAP1. On the other hand, increasing the amount of WT-GCAP1 with respect to E111V-GCAP1 led to a partial restoration of the Ca^2+^-regulation of GC1 enzymatic activity, although a 3-fold excess of WT was not enough to restore a fully normal behavior. In order to predict the dysregulation of the Ca^2+^ and cGMP homeostasis in a rod cell under conditions mimicking different expression levels of mutated GCAP1, we transferred the quantitative results from the enzymatic assays to a comprehensive model of mouse phototransduction that showed capable of reproducing photoresponses under both dim and bright stimuli^36,37^. We observed a crucial role for GCAP2 in mouse in compensating the dysregulation induced by the disease-associated E111V-GCAP1 which cannot possibly occur in human cones due to the lack of activity on GC1.

## Results and Discussion

Regulation of the GC1 activity by Ca^2+^ via GCAPs represents an exemplary case, in which an enzyme (GC1) that *per se* is not sensitive to Ca^2+^ can be either inhibited or activated by the same molecule (GCAP) depending on the subtle changes in intracellular [Ca^2+^]. The discovery of the highly cooperative negative feedback mechanism mediated by Ca^2+^ on the GC1 dates back to the late 80’s^38^, and yet the fine mechanisms of its regulation are not completely understood. It has been established that the GCAP1/2-mediated Ca^2+^ feedback on GC1 activity is the only one that occurs at very dim light intensities, corresponding to the single photon response^39^. By integrating quantitative information arising from assays performed with recombinant systems and numerical simulations, we sought to clarify some mechanisms that are apparently perturbed in cases such as the severe CORD form recently associated with the E111V mutation in GCAP1^10^. To investigate the role of each GCAP variant, namely WT-GCAP1, E111V-GCAP1 and WT-GCAP2 in GC1 (dys)regulation, we performed biochemical assays both in the presence of individual GCAPs and in the co-presence of the variants, and measured quantitative parameters to describe the Ca^2+^-dependence of the cGMP synthesis by GC1 and the apparent cooperativity of the process.

### Human GCAP2 does not activate human GC1

**Figure 1:**
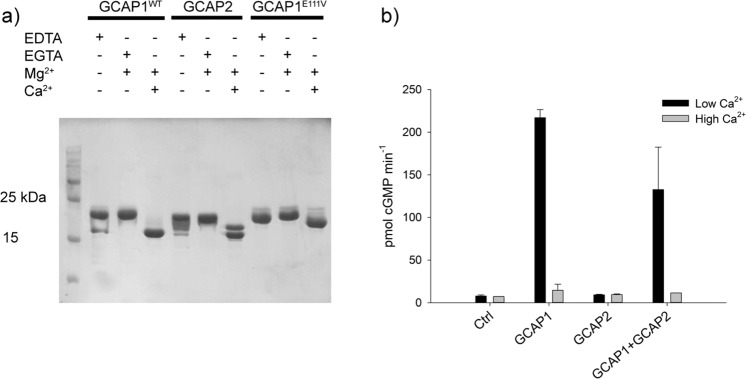
Purity of recombinant proteins, Ca^2+^-induced gel shifts and GC1 enzymatic assays. (**a**) SDS-PAGE of WT/E111V GCAP1 and GCAP2 in the presence of 5 mM EDTA, 4 mM EGTA + 1.4 mM Mg^2+^ and 1 mM Mg^2+^ + 4 mM Ca^2+^. Twenty micromolar WT-GCAP1, E111V-GCAP1 and WT-GCAP2 were incubated for 10 min at 30 °C in the presence or absence of ions and loaded in a 15% SDS-PAGE. Gel was Coomassie blue-stained. (**b**) GC1 enzymatic assays in the presence of 10 μM GCAP1, 10 μM GCAP2 and equal amounts of both (5 μM GCAP1 + 5 μM GCAP2) in the presence of less than 19 nM Ca^2+^ (low Ca^2+^) or ~ 30 μM Ca^2+^ (high Ca^2+^). Each bar represents the average of three replicas ± standard deviations (n = 3). Full-length gels are reported in Supplementary Fig. S1.

Human GCAP1 (both WT and carrying the E1111V mutation) and GCAP2 were heterologously expressed and purified at high purity levels. All GCAP variants responded to Ca^2+^ by switching to a different conformation that showed the typical increase in electrophoretic mobility (Fig. 1a), as observed in other calcium sensors^40^. The presence of smeared or multiple bands in SDS-PAGE gels is usual for calcium sensor proteins, and is related to the residual capability of the protein to bind Ca^2+^ in spite of the detergent-induced denaturation and the chelator present in the buffer^12^. Human GC1 was stably expressed in HEK cells and it concentrated in the membrane milieu (Fig. S1b), thus allowing the correct *in vitro* reconstitution of the GCAP-GC1 complex for biochemical assays.

While WT-GCAP1 was able to activate GC1 at low Ca^2+^, as observed in previous studies^10,11^, GCAP2 was unable to exert any regulation of GC1 as in both low and high Ca^2+^ conditions the levels of cGMP synthesis were unaltered with respect to those of the control (Fig. 1b). When the enzymatic assays were performed with equal amounts of GCAP1 and GCAP2, at low Ca^2+^ a slight decrease in the GC1 activation was observed (n = 3, p = 0.045) with respect to the sole presence of GCAP1, thus indicating that the presence of GCAP2 only slightly interferes with the activity of GCAP1, which was still capable of regulating GC1. No GCAP2-induced activation of GC1 was observed even when the experiments were performed at higher (12 mM) Mg^2+^ (Fig. S2), thus indicating that the inability is intrinsic and not due to a low amount of Mg^2+^-bound GCAP2 in the assay.

The finding that human GCAP2 does not activate human GC1 might seem surprising, but in fact it is in line with previous work indicating that in human retinas, and similarly in monkey, GCAP2 localizes in cone inner segments, soma and synaptic terminal and only at very low levels in inner segments^41^. GCAP2 is crucial for maintaining the integrity of the photoreceptor synaptic terminal, likely mediating the effect of light on the morphological remodeling changes of synaptic ribbons^42^ and even a single phosphorylation of GCAP2 has been shown to cause the complete retention of the protein in the inner segment^43^. Thus, while experiments with GCAPs^-/-^ transgenic mice clearly established a role for GCAP2 in regulating the GCAP1-dominated GC1 activity^39,44^, and thorough *in vitro* investigations confirmed such capability^7^, in humans GCAP2 might have not-yet clear functions non-related with phototransduction.

### CORD-associated E111V-GCAP1 is dominant and leads to GC1-dysregulation

**Figure 2:**
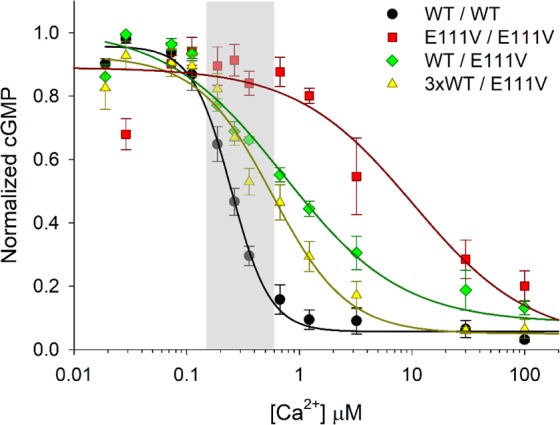
Ca^2+^-dependence of GC1 activity in the presence of various GCAP1 variants. The GC1 activity as function of [Ca^2+^] was measured in the presence of 10 μM WT-GCAP1 (black circles), 5 μM WT-GCAP1 + 5 μM E111V-GCAP1 (green diamonds) and 15 μM WT-GCAP1 + 5 μM E111V-GCAP1 (yellow triangles). E111V-GCAP1’s activity profile is referred to the published data^10^. Each data set is relative to 4–6 replicas and data are normalized according to both total protein content in membranes and maximum and minimum GC1 activity recorded in each replica; error bars represent s.e.m. Solid lines represent the results of data fitting while the grey box represents the physiological Ca^2+^ fluctuations in rod photoreceptors.

Since GCAP2 did not contribute to the activation of human GC1, we monitored the Ca^2+^-dependent regulation of the cyclase in the co-presence of different amounts of WT-GCAP1 and E111V-GCAP1, with the goal to quantitatively assess the effects of the two variants on the same enzymatic target. Over the relatively narrow window of physiological variations of intracellular [Ca^2+^] (∼10 nM–600 nM), WT-GCAP1 correctly switched from GC1-inhibitor to GC1-activator (Fig. 2, black curve). The IC_50_ value, i.e. the [Ca^2+^] at which the synthesis of cGMP and therefore the activity of GC was half-maximal, was 251 ± 19 nM (n = 6) and the cooperativity of the process was high (h_c_ = 2.60 ± 0.42; n = 6; Table 1).

**Table 1:** Results from enzymatic assays. Human GC1 activity as a function of free [Ca^2+^].

	GCAP1^WT^ (n = 6)	GCAP1^E111V b^ (n = 4)	GCAP1^WT/E111V^ (n = 6)	3XGCAP1^WT^/GCAP1^E111V^ (n = 6)
IC_50_ (nM)	251 ± 19^a^	(10 ± 5)x10^3^	694 ± 75	497 ± 49
h_c_	2.60 ± 0.42	0.83 ± 0.27	0.87 ± 0.18	1.49 ± 0.35

^(a)^ reported data are mean ± s.e.m.

^(b)^ data from. ref. ^10^.

Like many other COD/CORD-associated GCAP1 mutants, E111V-GCAP1 showed a dramatic shift of GC1 regulation to very high Ca^2+^ levels, with IC_50_^E111V^ = 10 ± 5 μM (n = 4) and a significantly lower cooperativity (h_c_ = 0.83 ± 0.27, n = 4; see red curve in Fig. 2)^10^ compared to the WT, thus constitutively activating the GC1 target over a physiological range of [Ca^2+^]. Those biochemical assays, however, were performed in the sole presence of E111V-GCAP1. When the assay was performed by mixing equal amounts of WT and E111V-GCAP1, thus resembling the heterozygous WT/E111V case found in CORD patients, the situation led to a different scenario (Fig. 2, green curve). While no significant change in the cooperativity could be observed (Table 1), the IC_50_ value shifted down to 694 ± 75 nM (n = 6) (Fig. 2, Table 1). Although this value is still insufficient to inhibit the GC1 at physiologically high [Ca^2+^], leading to ∼55% of the maximal activity, it is definitely lower compared to the ∼90% activity of E111V-GCAP1 alone.

WT-GCAP1 is thus capable of partly restoring the functional switch of GC1 in the presence of a mutant that would otherwise block the enzyme in a constitutively active state. A similar result was previously obtained by Dizhoor and co-workers^21^, who investigated in a bovine system the Y99C-GCAP1 variant associated with COD, and noticed that enzymatic assays in the co-presence of WT and mutated GCAP1 still led to constitutive GC1 activity. The authors also investigated the co-presence of Y99C-GCAP1 and WT-GCAP2, obtaining very similar results, and thus concluded that the mutant stimulates the cyclase in the presence of equimolar concentrations of either WT-GCAP1 or GCAP2^21^. However, those assays were performed in a bovine system, where GCAP2 plays a role in GC1 activation, at odds with our current findings referring to a human system.

Since human GCAP2 does not activate GC1, we sought to investigate whether the observed partial restoring capability of WT-GCAP1 to restore GC1 activity in the presence of E111V-GCAP1 could even increase in the presence of extra WT-GCAP1. The assays were performed with 3-fold excess of WT-GCAP1, while keeping the concentration of E111V unaltered with respect to the previous conditions. Interestingly, a statistically significant decrease of IC_50_ was observed (497 ± 49 compared to 694 ± 75; n = 6; one-tailed p = 0.029; Table 1). Although the residual activity of GC1 at physiologically high [Ca^2+^] was still quite high (∼45%; Fig. 1, yellow curve) the regulation profile became more cooperative, as shown by the increase in h_c_ (1.49 ± 0.35 vs. 0.87 ± 0.18, n = 6). Therefore, although the switch was still non-optimal, the presence of 3-fold excess WT-GCAPs seems to slightly attenuate the dominant and detrimental effect caused by the E111V-GCAP1 mutation.

The observed results of GC1 activity in the presence of different amounts of GCAP1 and its E111V variant are not straightforward to interpret. Human WT-GCAP1 and E111V-GCAP1 have very similar apparent affinity (EC_50_) for GC1^10^, therefore it seems unlikely that the cause of the still dominant effect of the mutant in a situation of 3-fold excess of WT be attributed to the preferential formation of a complex with the GC1. A more realistic explanation could be the existence of different equilibria between the oligomeric states of WT-GCAP1 and E111V-GCAP1, which could result in different amounts of monomeric GCAP available for GC regulation. GCAP1 is known to form dimers^10,45^ and under conditions mimicking the physiological ones E111V-GCAP1 was also found to be dimeric^10^. We cannot exclude that in the conditions of our *in vitro* assays the tendency of WT-GCAP1 to form dimers enhanced at the higher concentrations corresponding to the 3-fold excess experiments, *de facto* limiting the availability of monomers that could further improve the restoration of the GC1 regulation towards a physiological behavior.

### Expression levels of normal GCAPs set the homeostasis of Ca^2+^ and cGMP in a computational model of E111V-GCAP1 mouse rod

In order to evaluate the putative cellular consequences of the Ca^2+^/cGMP dysregulation brought about by the E111V-GCAP1 mutation associated with CORD and the extent of a potential compensation by normal GCAPs, we used the parameters experimentally measured in the human system to predict the rate of cGMP synthesis in a mouse rod illuminated by flashes of increasing intensity. We used a comprehensive kinetic model of phototransduction that demonstrated very effective in reproducing experimental results from dim to bright light conditions^37^. In particular, we could directly probe the role of each GCAP, namely WT/E111V GCAP1 and GCAP2, in regulating the levels of second messengers by virtually tuning the contribution of each protein to GC1 regulation and simulating the dark-adapted state of the cell as well as the response to specific light stimuli, thus investigating the dynamic shaping of cGMP synthesis.

The significant perturbation of the GC1 regulation induced by the E111V mutation in GCAP1 assessed *in vitro* was reflected by a substantial increase of the dark levels of Ca^2+^ and cGMP (Table 2).

**Table 2:** Increase in dark levels of Ca^2+^ and cGMP according to numerical simulations of a mouse rod outer segment compared to a wild-type case.

	GCAP1^WT/E111Va^	GCAP1^E111V/E111V^	3XGCAP1^WT^/GCAP1^E111V^ + GCAP2	3XGCAP1^WT^/GCAP1^E111V^-GCAP2
X-fold [Ca^2+^_free_]	3.1	12.5	1.4	3.0
X-fold [cGMP]	1.4	2.0	1.1	1.4

^a^combination of individual weight factor f_i_ to simulate each condition are illustrated in the Methods.

The simulation of the rod in the dark for a heterozygous WT/E111V mouse resembles the case of adCORD pattern in humans, in which half of the GCAP1 pool is made of WT molecules and the other half carries the E111V mutation. Under these conditions, a 3.1-fold increase in the level of intracellular Ca^2+^ and 1.4-fold increase in that of cGMP was predicted by numerical simulations (Table 2). These results are substantially in line with the experimental measurements of intracellular [Ca^2+^] in Y99C-GCAP1 mice, where an increase of 1 to 2-fold depending on the expression levels of the mutant over a background of WT-GCAP1 was observed^26^. To probe the effects of different expression levels of mutant vs. WT-GCAP1, we performed numerical simulations of other putative cases, first one in which the whole pool of GCAP1 carried the E111V mutation in the co-presence of endogenous WT-GCAP2. The simulated increase of dark levels of Ca^2+^ and cGMP was dramatic in this hypothetical E111V/E111V homozygosis case (12.5-fold and 2.0-fold, respectively; Table 2). In line with our *in vitro* experiments with human proteins, we also simulated the effects of a 3-fold excess of WT-GCAP1 over a background of constant E111V-GCAP1, both in the presence and in the absence of endogenous GCAP2. Interestingly, extra-delivery of WT-GCAP1 in the presence of GCAP2 restored an almost WT-like homeostasis of dark Ca^2+^ and cGMP (1.4-fold and 1.1. fold, respectively; Table 2). However, the same delivery of WT-GCAP1 performed in the absence of endogenous GCAP2 did not restore a WT-like homeostasis for second messengers, and led to a dramatic increase of 3.0-fold dark Ca^2+^ and 1.4-fold cGMP, similar to what was observed in the simulated heterozygous WT/E111V case (Table 2). Hence, simulations suggest that GC1 activation by GCAP2 is fundamental in mouse photoreceptor to set the normal levels of second messengers. In its absence, the normally dominant WT-GCAP1 although delivered in excess cannot compensate for the detrimental dysregulation of GC1 by the pathogenic E111V mutation.

### GCAP2 shapes cGMP synthesis in E111V-GCAP1 mouse photoresponses

**Figure 3:**
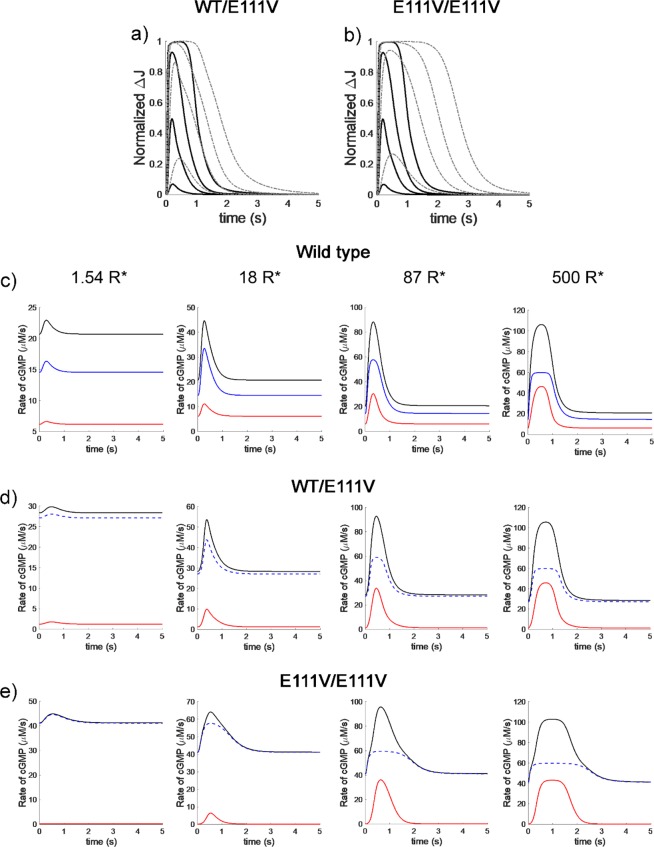
Simulation of flash responses and dynamics of cGMP synthesis in a mouse rod, from dim to bright light conditions. After equilibration of the rod outer segment in the dark, 24 ms flashes were delivered, leading to 1.54, 18, 87 and 500 photoisomerizations of rhodopsin; (**a**,**b**) Black traces: WT GCAPs-containing rods. Dash-dotted gray lines: photoresponses in a WT/E111V heterozygous case (**a**) and E111V/E111V homozygous case (**b**). Photocurrents represent the suppression of the dark current at each light intensity and have been normalized**. (c**–**e**) Simulated time course of the rate of cGMP synthesis by GC1 in the same conditions as in (**a**,**b**). Blue lines: contribution by WT-GCAP1 + E111V-GCAP1 (if present). Red lines: contribution by GCAP2. Black lines: overall GCAPs contribution obtained by adding up the former terms.

Perturbation of the second messenger homeostasis in the dark might lead to alterations in the photoresponse of the affected cells. To probe such potential variations, we simulated photoresponses from mouse rods expressing the same amounts of GCAPs investigated in the dark following the excitation of the phototransduction cascade by flashes of increasing intensity, from dim to saturating. The resulting dynamics of suppression of the dark current in a rod of a WT mouse and the respective cases for a heterozygous WT/E111V and a homozygous E111V/E111V-GCAP1 case are reported in Fig. 3a,b, respectively.

For all the tested flash intensities the photoresponses of the mutant cases were prolonged compared to the WT, with increased time to peak. The prolongation of the photoresponse apparently depends on the relative abundance of E111V-GCAP1 with respect to the WT and was in fact significantly more pronounced in the homozygous (Fig. 3b) compared to the heterozygous case (Fig. 3a). Similar results were experimentally measured in Y99C-GCAP1 mice^26^, although the effect was less prominent, probably due to the milder phenotype that developed COD and not CORD as in the case of E111V-GCAP1.

Our computational implementation of the GC1 regulation by different GCAP variants allowed us to ideally dissect their individual contributions in shaping the rate of cGMP synthesis at different light stimuli. Figure 3c,d reports on the overall GCAPs contribution (black line) to the time course of cGMP synthesis as a sum of the contribution by GCAP1 variants (blue line) and WT-GCAP2 (red line). Clear differences could be observed between WT and WT/E111V heterozygous case. For WT rods, increasing the flash intensity led to an increasing contribution of GCAP2 in shaping the cGMP synthesis rate, which was less important for very dim flashes, but became predominant at bright intensities (Fig. 3c**)**, in line with the Ca^2+^-relay mechanism^3,35^. In the case of WT/E111V heterozygous mouse, however, the unbalance in the contributions became apparent under dim light conditions, where GCAP1 variants dominated, while GCAP2 was still capable of shaping the rate at brighter flashes (Fig. 3d). It should be noticed that, in spite of the dramatic perturbation in the dark levels of Ca^2+^ ad cGMP (Table 2), our model predicts that the photoreceptor would still respond to light stimuli, with all in all minor perturbation of the photoresponse shape (Fig. 3a), thus fully in line with experimental observations^26^.

The major dysregulation of second messenger homeostasis observed in the hypothetical E111V/E111V homozygous case, where the whole GCAP1 pool carried the E111V mutation reflected in a dramatic perturbation of the photocurrent shapes, which became significantly prolonged and did not completely shut-off even after 5 s for brighter stimuli (Fig. 3b). Under dim flashes, GCAP2 did not contribute at all to the shaping of the cGMP synthesis rate, but at middle to bright light conditions, where GCAP1 was almost completely blocked in the GC1-constitutively activating state, GCAP2 provided most of the dynamic contribution (Fig. 3e).

**Figure 4:**
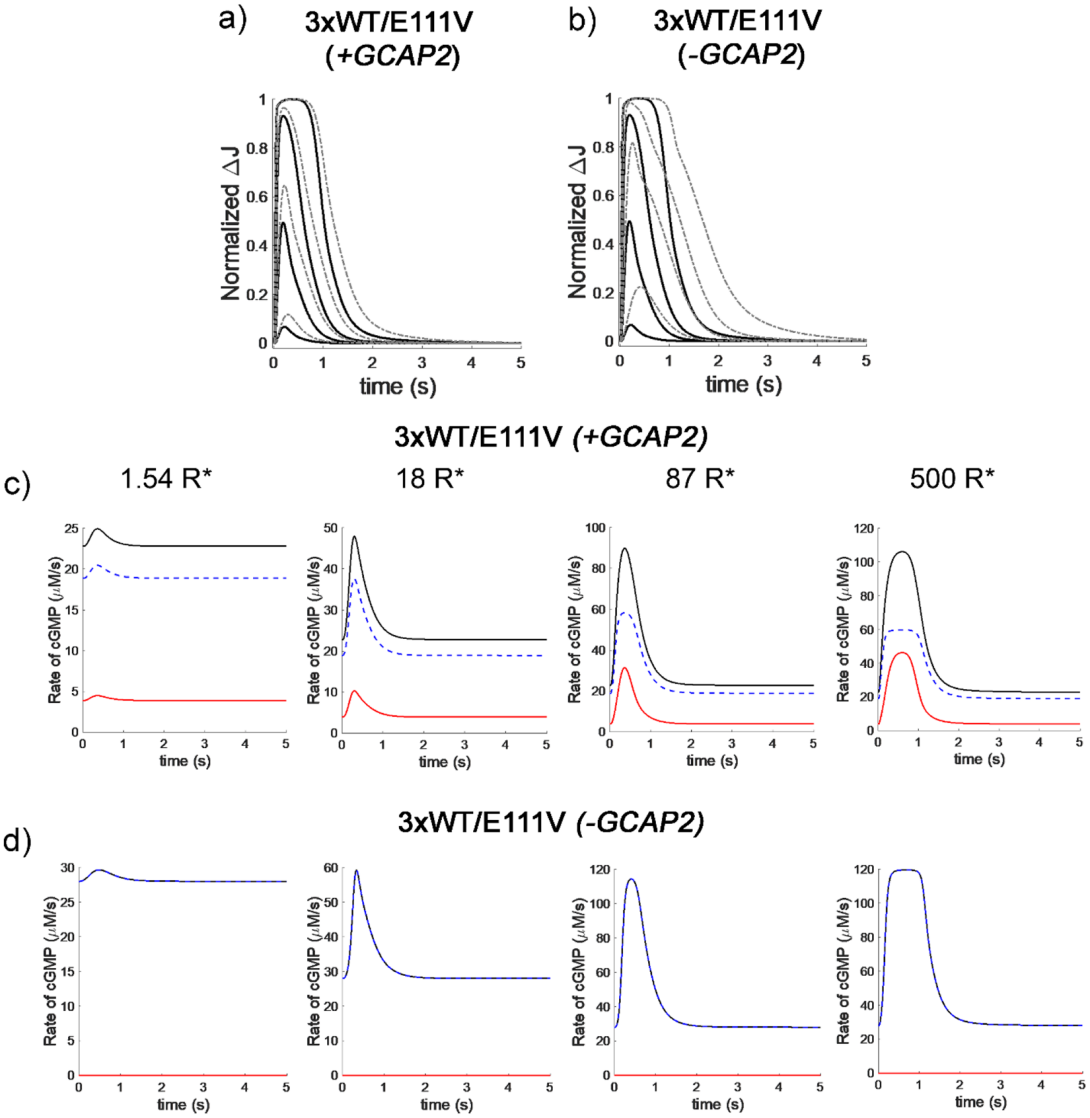
Simulation of flash responses and dynamics of cGMP synthesis in a mouse rod, from dim to bright light conditions, in the presence of 3-fold WT-GCAP1 with respect to E111V-GCAP1. Panels (**a**,**c**) refer to simulations in which endogenous GCAP2 was present. Panels (**b**,**d**) refer to simulations in the absence of endogenous GCAP2. All simulated conditions and symbols are the same of Fig. 3.

Simulation of 3-fold WT-GCAP1 delivery over a background of E111V-GCAP1 variant suggested a scenario that strictly depends on the presence of the other endogenous GCAP2. Indeed, when endogenous GCAP2 was considered in the simulations, the mutant-induced prolongation of the photocurrent was reduced at all light intensities (Fig. 4a) and the dynamics of cGMP synthesis as well as the specific contributions of GCAPs (Fig. 4c) were overall fairly similar to the WT case (Fig. 3c). This suggests that under these conditions it would be in principle possible to partly restore a normal shaping of both the photocurrent and cGMP synthesis profile, thus minimizing the perturbation of the second messengers (Table 2). However, when the same delivery of 3-fold WT-GCAP1 was simulated in the absence of endogenous GCAP2, the prolongation of the photocurrents became significant under all light regimes (Fig. 4b), resembling that of heterozygous WT/E111V (Fig. 3a). Nevertheless, in this case the absence of GCAP2 led to a completely GCAP1 (WT and E111V)-driven cGMP synthesis, with higher maximal levels of synthesis at each light intensity (Fig. 4d) compared to the heterozygous case (Fig. 3d). Hence, in spite of the dominant role of this GCAP1 isoform in the WT photoreceptor, the excess of GCAP1 alone is not sufficient to compensate for the absence of GCAP2 in mouse rods.

The crucial role of GCAP2 in compensating for the extreme perturbations of cGMP signaling induced by blocking GCAP1 into a GC1-constitutively active state was proposed by us for a bovine system based on comprehensive model of the phototransduction kinetics^46^ and was then experimentally confirmed in a mouse cone system^34^.

## Conclusions

In conclusion, our findings support the view that the severity of COD/CORD phenotypes associated with point mutations in *GUCA1A* correlates both with the dysregulation of the cGMP signaling induced by the point mutation and with the levels of mutated GCAP1 over WT-GCAPs. Increasing the levels of WT-GCAP1 on a background of disease-associated mutants may partly attenuate the increased dark levels of Ca^2+^ and cGMP, which are ultimately associated to cell death^47,48^ and reduce the perturbation of the photocurrent dynamics. Since promising strategies for the delivery of WT recombinant proteins to photoreceptors are under development^49,50^, our findings might be relevant for future protein therapies aimed at slowing retinal degenerations associated with *GUCA1A* mutants. However, our data also show that, while useful for the purpose, extra-WT-GCAP1 alone would probably not be sufficient to restore a normal regulation of GC1 in human retinas. The intricate Ca^2+^-relay mechanism involving GC1 and the GCAPs might be substantially species-dependent, and whether other regulators in human photoreceptors may mimic the synergic activation of GCAP1 and GCAP2 on GC1 observed in mouse and bovine retinas remains to be clarified.

## Materials and Methods

### GCAPs expression and purification

Synthetic genes (Genscript) corresponding to the cDNA of human GCAP1 (Uniprot: P43080) and GCAP2 (Uniprot: Q9UMX6) were cloned into pET-11a(+) vectors using NdeI and NheI as restriction site. Mutagenesis, protein expression and purification of myristoylated proteins were performed as described previously^10,12^. Briefly, cells were let grown at 37 °C until an OD_600_ value of 0.4 was reached, then myristic acid (100 μg/mL, in 50% EtOH, pH 7.5) was added. After the induction with 1 mM IPTG, cells were let grown for 4 hours at 37 °C. Both WT and E111V-GCAP1 as well as GCAP2 were purified from inclusion bodies. After protein denaturation by 6 M guanidinium hydrochloride and refolding steps by dialysis, two subsequent chromatographic steps (size exclusion and anionic exchange chromatography) were performed. Proteins purity was tested by Polyacrylamide Gel Electrophoresis in the presence of Sodium-Dodecyl-Sulfate (SDS-PAGE) and was found to be at least 95% (see Fig. 1a). The efficiency of protein myristoylation was proven by HPLC and mass spectrometry. After buffer exchange (20 mM TRIS, 150 mM NaCl, 1 mM DTT), proteins were shock frozen and stored at −80 °C until use.

### GC expression and enzymatic assays

A stable cell line expressing human guanylate cyclase isoform 1 (GC1) was obtained as explained previously^10,51^ by transfecting HEK293 flp cells with pIRES-eGFP plasmid using Turbofect reagent. Transfected clones were selected based on the resistance to geneticin (G418). Cells were cultured in DMEM media containing fetal bovine serum (10% v/v), streptomycin (100 μg/mL), penicillin (100 units/mL) and geneticin (500 μg/mL). At 90% confluence the cells were harvested, washed with sterile PBS and stored at −80 °C. To perform enzymatic assays on isolated membranes cells pellets were suspended in lysis buffer (10 mM HEPES pH 7.4, 1 mM DTT, protease inhibitor cocktail 1X) and incubated for 20 min on ice. After 15 up-and-down cycles with a 1 mL syringe on ice, cells were centrifuged for 20 min at 10000 rpm. The obtained pellets were suspended in 50 mM HEPES pH 7.4, 50 mM KCl, 20 mM NaCl, 1 mM DTT.

First, GC1 activity was assessed incubating 10 μM GCAP1 or GCAP2 or a combination of both (5 μM GCAP1 + 5 μM GCAP2) in the absence (<19 nM) and in the presence (~30 μM) of Ca^2+^, obtained using 10 mM K_2_H_2_EGTA and K_2_CaEGTA buffers^51^ respectively (Fig. 1b**)**. Basal GC1 activity was assessed as internal control by replacing GCAPs with equal amount of the buffer used for protein storing. Then, the enzymatic activity as a function of [Ca^2+^] was measured as in previous work^10^, with minor changes explained in the following. Several combinations of GCAPs were tested, namely: (i) 10 µM WT-GCAP1; (ii) 5 μM WT-GCAP1 + 5 μM E111V-GCAP1; iii) 15 μM WT-GCAP1 + 5 μM E111V-GCAP1. Data for E111V-GCAP1 alone are referred to our previous publication^10^. Ca^2+^ concentration in each assay was carefully determined by combining the same K_2_H_2_EGTA and K_2_CaEGTA buffers previously mentioned. The assay consisted in incubating for 5 min at RT the diluted proteins with a fixed amount of extracted membranes, then reaction buffer (30 mM MOPS pH 7.2, 20 mM KCl, 10 mM, 4 mM NaCl, 1 mM DTT, 3.5 mM MgCl_2_, 1 mM GTP, 300 μM ATP, 160 μM Zaprinast) was added and the samples were incubated for 5 min at 30 °C. The reaction was blocked by the addition of 50 mM EDTA and incubating at 98 °C for 5 min. After centrifugation at 13000 rpm the supernatant was loaded in a C18 reverse phase column (Chromolith column, Millipore) previously equilibrated with 5 mM KH_2_PO_4_. Following a previously established protocol^10^ on an HPLC apparatus (Jasco) the cGMP concentration was determined by acetonitrile gradient. Four to 6 repetitions of each assay were performed. Data have been normalized to the total protein content present in membranes used for the assay (amido black, Sigma) and on the minimum and maximum GC-activity. IC_50_ and Hill coefficient (h_c_) values were determined for each assay by curve fitting to a 4-parameter Hill function (Sigma Plot 12.5) and mean, standard deviation and standard error of the mean (s.e.m.) were determined. All data distributions passed the Shapiro-Wilk normality test and the observed parameters IC_50_ and h_c_ were compared with one another by one-tailed t-test (SigmaPlot 12.5), rejecting in all cases the null hypothesis (p < 0.05).

### Numerical simulations of mouse photoresponses

A previously developed comprehensive biochemical model of phototransduction in mouse rods was used for numerical simulations^37^. All parameters describing the phototransduction cascade were left unaltered, the only difference being the way the reaction describing the dynamic synthesis of cGMP by GC1 was implemented. In the present implementation, to account for specific contributions of GCAP variants to the cGMP synthesis in a mouse photoreceptor, each variant (GCAP_i_) was described to contribute by a specific fraction *f*_*i*_ to the maximal activation (*α*_*max*_) of GC1:

$$\frac{d[cGMP]}{dt}=\sum _{i}\frac{{f}_{i}\times {\alpha }_{max}}{1+{(\frac{C{a}_{free}^{2+}}{I{C}_{{50}_{i}}})}^{{h}_{{c}_{i}}}}\,{\rm{where}}\,\sum _{i}{f}_{i}=1$$

The value for *α*_*max*_, assumed to be the same for each variant, was fixed to 120 μM/s, as in the previous model, while *IC*_50_ and *h*_*c*_ for WT-GCAP1 and GCAP2 were fixed to their experimentally measured values in mouse rod outer segments^7^. *IC*_50_ and *h*_*c*_ for E111V-GCAP1 were set to their relative values (-fold variation) compared to the wild type, according to the parameters experimentally measured for the human orthologs (Table 1). The use of different weight factors *f*_*i*_ is a convenient way to account for the specific contribution of each GCAP variant to the overall regulation of GC1, with the assumption of a similar apparent affinity between GCAP variants and GC1, so that the variants may compete for the target based solely on their concentration/expression levels. This condition has been demonstrated to be realistic for E111V and WT human GCAP1, for which substantially unaltered EC_50_ values for human GC1 were measured^10^, as well as for murine GCAP1 and GCAP2, which showed almost identical EC_50_ values for murine GC1 in rod outer segment membranes^7^.

With the above assumptions, by tuning the *f*_*i*_ terms one can simulate a broad variety of experimental conditions; for example, the situation of a heterozygous mouse E111V/WT, corresponding to the human adCORD case, in which half of the GCAP1 molecules are WT and half carry the E111V mutations, in the presence of normal amounts of WT GCAP2 was modeled as follows:

$$\begin{array}{ccc}\frac{d[cGMP]}{dt} & = & \frac{d{[cGMP]}^{GCAP{1}^{WT}}}{dt}+\frac{d{[cGMP]}^{GCAP{1}^{E111V}}}{dt}+\frac{d{[cGMP]}^{GCAP{2}^{WT}}}{dt}\\  & = & {\alpha }_{max}\times (\frac{0.25}{1+{(\frac{C{a}_{free}^{2+}}{I{C}_{{50}_{GCAP{1}^{WT}}}})}^{{h}_{GCAP{1}^{WT}}}}+\frac{0.25}{1+{(\frac{C{a}_{free}^{2+}}{I{C}_{{50}_{GCAP{1}^{E111V}}}})}^{{h}_{GCAP{1}^{E111V}}}}+\frac{0.5}{1+{(\frac{C{a}_{free}^{2+}}{I{C}_{{50}_{GCAP{2}^{WT}}}})}^{{h}_{GCAP{2}^{WT}}}})\end{array}$$

The case of homozygous mouse E111V/E111V in the presence of endogenous GCAP2 was simulated by setting f_GCAP1_^WT^ = 0; f_GCAP1_^E111V^ = 0.5; f_GCAP2_ = 0.5. Finally, the case of 3-fold excess of WT-GCAP1 over a background of E111V-GCAP1 and endogenous GCAP2 was obtained by setting: f_GCAP1_^WT^ = 0.375; f_GCAP1_^E111V^ = 0.125; f_GCAP2_ = 0.5, while the same excess in the absence of endogenous GCAP2 was simulated by: f_GCAP1_^WT^ = 0.75; f_GCAP1_^E111V^ = 0.25; f_GCAP2_ = 0. All numerical simulations were performed in Matlab as explained in earlier works^36,37^.

## Supplementary information

Supplemental Information
