# Supplementary material for: Normal GCAPs partly compensate for altered cGMP signaling in retinal dystrophies associated with mutations in GUCA1A
Source: Sci Rep. 2019 Dec 27;9:20105. doi: 10.1038/s41598-019-56606-5 (PMC6934868; doi:10.1038/s41598-019-56606-5)
Supplement: Supplementary file 1 — Supplemental Information [file 41598_2019_56606_MOESM1_ESM.pdf]

Supporting information

Normal GCAPs partly compensate for altered cGMP signaling in retinal dystrophies associated with mutations in GUCA1A

Daniele Dell'Orco[1*] and Giuditta Dal Cortivo[1]

[1]Department of Neuroscience, Biomedicine and Movement Sciences, Section of Biological Chemistry, University of Verona, I-37134 Verona, Italy

*to whom correspondence should be addressed. Telephone: +39-045-8027637; E-mail: daniele.dellorco@univr.it

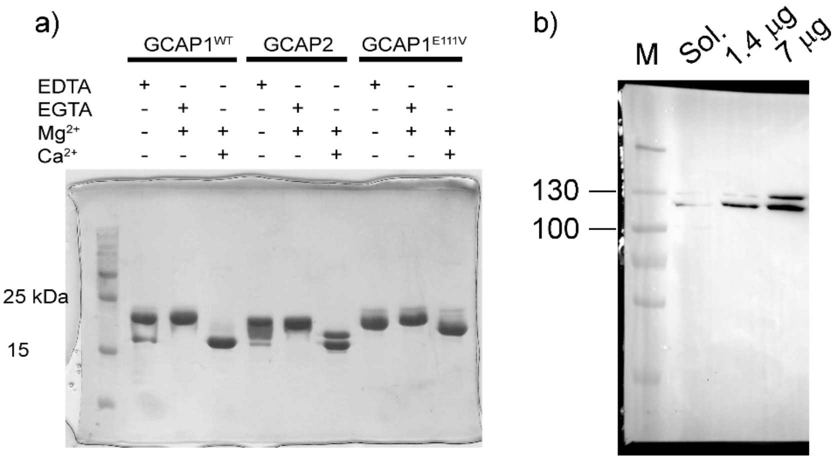

Figure S1. Purity of recombinant proteins and GC1 enzymatic assay. a) Full-length gel used for the preparation of Figure 1a. b) Western blot analysis of HEK293 soluble phase (Sol) and two different amounts (1.4 and 7 µg) of membrane proteins previously quantified by amido black. Anti-GC1#3 antibody (Ref. 51) was used (1:1000 dilution).

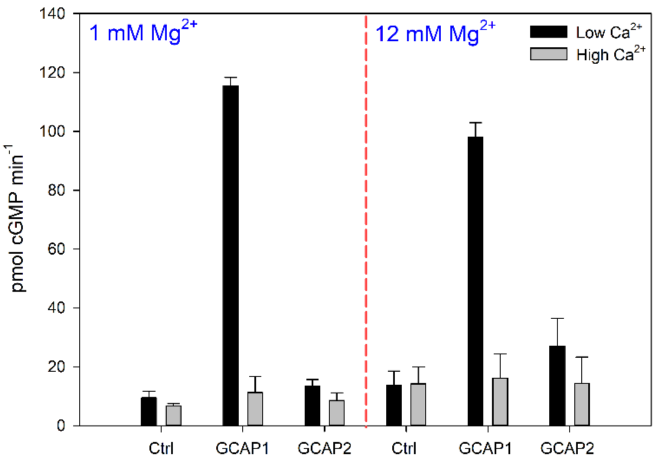

Figure S2: GC assay at different Mg^{2+} concentrations. Guanylate Cyclase activity induced by GCAPs was analyzed at 1 mM (left) and 12 mM Mg^{2+} (right), in the presence of low (< 19 nM) and high (~30 µM) Ca^{2+} concentration. Each measurement represents the average of three replicas.
